# Revision Stapes Surgery

**DOI:** 10.1007/s40136-021-00379-x

**Published:** 2022-01-24

**Authors:** Hitomi Sakano, Jeffrey P. Harris

**Affiliations:** 1 Department of Otolaryngology, University of Rochester, Rochester, NY, USA; 2 Department of Otolaryngology-Head & Neck Surgery, University of California San Diego, San Diego, CA, USA; 3 Department of Surgery, Division of Head and Neck Surgery, Veterans Affairs Hospital, San Diego, La Jolla, CA, USA

**Keywords:** Stapedotomy, Stapedectomy, Revision stapes surgery, Incus erosion, Stapes prosthesis, Otosclerosis

## Abstract

**Purpose of Review:**

This review briefly covers the history of stapedectomy, discusses the indications and problems encountered with revision surgery, and provides case examples with solutions.

**Recent Findings:**

Revision surgery is challenging and successful outcome even in the most experienced specialists is 45–71%, which is far less than that of primary surgery.

**Summary:**

Careful evaluation of the reasons for reoperation, anticipation of the common problems, and patient education on reasonable expectations are all very important for success.

## Introduction

Stapedectomy is performed primarily for the condition of otosclerosis in which there is ankyloses of the stapes, first described by Joseph Toynbee who studied 1659 temporal bones [1, 2]. The condition is histologically characterized by resorption and deposition of bone at the fissula ante fenestra in 80% of cases [3]. Clinically, it may be observed as a “Schwartze’s sign,” a reddish hue behind the tympanic membrane as described by Herman Schwartze 1873 due to increased vascularity of the cochlear promontory [4]. Disease progression can then lead to fixation of the stapes footplate, causing conductive hearing loss, typically with a Carhart’s notch where there is a tendency toward closure of the air–bone gap (ABG) at 2000 Hz [5]. Less frequently, there can be involvement of the endosteal layer of the otic capsule which can cause sensorineural loss as well. Histological prevalence of otosclerosis has been reported to range 2.5 to 8.3%, but clinically significant prevalence is estimated to be 0.3% [6].

The first otologist to mobilize and remove the stapes was Johannes Kessel in 1876 [3]. Although many attempts were made thereafter, due to complications and even deaths in the 1900s, these operations were shunned by prominent otologists such as Adam Politzer and Friedrich Siebenmann and stapes surgery halted for many decades. Kessel was censored for unscrupulousness and ultimately retired embittered [7]. Later, Jenkin and Holmgren found that fenestration of the lateral semicircular canal would improve hearing. Sourdille then improved on this with a three-stage tympanolabyrinthopexy open approach [7]. Lempert then heard Sourdille’s lecture and developed a single-stage endaural fenestration approach. Rosen then resurrected the stapes mobilization. Inspired by Rosen and realizing the importance of sealing the oval window after reading a case report by Frederick Jack from the Mass Eye and Ear Infirmary, Shea improved the stapedectomy procedure by utilizing a vein graft and polyethylene tubing fashioned into a prosthesis [7]. As stapedectomy procedure took off, Lempert’s career ended as he failed to adopt this technique.

### Primary Stapedectomy

Stapedectomy, as we know today, is an elegant procedure further refined after the initial description by Shea. Most procedures are approached transcanal with removal of sufficient scutum to expose the incudostapedial joint, the pyramidal process, and the tympanic portion of the fallopian canal. Many now choose to use laser for hemostasis and minimize the use of drilling on the footplate, although the surgery can be performed without laser or a combination of both. The stapedial tendon is cut, the incudostapedial joint disarticulated, the posterior crus of the stapes is weakened with the laser, and the stapes suprastructure downfractured toward the promontory. The footplate is either lasered in a rosette fashion, or the fenestration can be done with a low rpm (revolutions per minute) drill. A small-hole fenestration is performed of approximately 0.7 mm in diameter. There are various pistons that are placed within the stapedotomy and hooked onto the incus; the author uses a Teflon or fluoroplastic stapes piston with a nitinol Shepherd’s crook which is tightened with a pulse of a laser. In patients with a nickel allergy, nitinol should be avoided, and a prosthesis is chosen that must be crimped by hand. The overall risk of deafness is 1–2%, and overall success rate to an air–bone gap of ≤ 10 dB can be as high as 94% [8•].

### Revision Stapedectomy

Indications for revision surgery should be considered after a period of observation. Aside from intractable vertigo or facial nerve complication requiring immediate intervention, revision surgery is indicated primarily for persistent or recurrent conductive hearing loss of ≥ 20 dB. Because of the postoperative inflammation, it is best to wait at least several months before consideration of revision surgery. Recurrent hearing loss would suggest a problem that developed over time after initial successful surgery with air–bone gap closure (such as incus erosion), while persistent hearing loss after an otherwise straightforward surgery may suggest other conditions that also cause conductive hearing loss (such as a third window or ankylosed malleus). Preoperative computed tomography (CT) imaging should be considered in identifying the causes for symptoms (i.e., prosthesis displacement and incus long handle erosion). It may also reveal unanticipated causes of persistent conductive hearing loss, such as semicircular canal dehiscence (most commonly superior canal, but posterior canal opening into the jugular bulb should also be investigated) which are unmasked by an otherwise apparently successfully performed surgery [9]. CT can also overestimate the depth of penetration of the prosthesis into the vestibule, and thus, interpretation of CT must be taken cautiously [10].

As in initial stapedectomy, a transcanal approach is used. We prefer monitored anesthesia care (MAC) because having the patient awake for the manipulation of the prosthesis is valuable to assess vertigo. Endaural incisions may improve visualization if needed. The tympanomeatal flap is elevated. The chorda tympani nerve may be scarred and difficult to preserve. Lasers (CO_2_, KTP, or argon) may be helpful to lyse adhesions, though not necessary. The scutum is likely adequately open from prior surgery. By far, the most common site of failure is the prosthesis position with or without incus erosion [11–15]. Thus, the prosthesis position and oval window are carefully examined. Often, replacement of the initial prosthesis with a new one is necessary. However, checking the entire the ossicular chain for mobility is essential as bony fragments or unappreciated ankyloses of the malleus and incus may have caused the poor hearing result. If the patient is awake during the procedure, the hearing can be tested intraoperatively with a tuning fork and soft whisper. After reestablishment of the ossicular chain, the oval window should be sealed with tissue (fat or perichondrium) or blood, and the tympanomeatal flap is laid back down. The authors strongly prefer a tissue seal to prevent a persistent fistula.

### Problems Encountered in Revision Surgery

Immediately or over time, conductive hearing loss of greater than 20 dB may occur after initial stapedotomy or stapedectomy. Revision may be required in approximately 20% regardless of the method of primary stapedectomy or stapedotomy [16]. There are various findings that account for this, including (1) prosthesis displacement or malfunction, (2) necrosis of incus, (3) adhesions, (4) osseous closure of the oval window, (6) ankyloses of malleus or incus to the attic, and (7) reparative granuloma. The first two, individually or in combination, account for the vast majority of cases, 82% [17].

### Prosthesis Problem

Prosthesis malfunction is by far the most common reason for revision; it accounts for up to 60% of cases [11–15]. The majority are due to displacement out of or to the edge of the oval window, displacement at the wire attachment to the incus, or both [11, 13]. These problems are much more amenable to repair with a superior success rate overall compared to other problems described below [11]. Most often, the problem is traced to inappropriate prosthesis length (too short) chosen at the time of primary surgery. Change in prosthesis length between primary and revision surgeries is required in 73.5% of cases [18] with the vast majority (64–85%) of these requiring a longer prosthesis [12, 18]. Thus, measurement is an essential part of successful primary surgery. One explanation for this may be that surgeons may err on placing a shorter rather than longer prosthesis due to potential risk of vertigo with a longer prosthesis. However, one must account for a potential 0.5-mm displacement of the prosthesis once it is in place [19]. Thus, careful measurement and selection of the appropriate length of the prosthesis may avoid need for future revision surgery. A simple method to determine if the prosthesis is too short is to gently nudge the prosthesis from side to side, and if it is short, it will slide out of the fenestrated opening in the footplate.

### Erosion of the Incus

Erosion of the incus is the second most commonly seen cause for revision stapedectomy, ranging from 6.3 to 32% [11, 13, 15, 20, 21]. The incus long process is supplied by the incudal artery (arising from the ossicular branch of the anterior tympanic artery) which feeds the mucosal arteries and the nutrient foramen (within the incus body). The lenticular process is also supplied by anastomoses from the arteries of the stapes tendon and posterior crural artery [22]. Avascular necrosis has been thought to be caused by compromised blood supply by a tightly crimped wire. However, Schuknecht challenged this idea stating “the incus contains a central nutrient vessel and a network of mucosal vessels adequate to supply the tip of the long process” [23]. A recent study showed that most nutritive foramina are found in the upper two-thirds of the long process along the anteromedial aspect where it is unlikely to be affected by a crimped prosthesis [24]. Incus necrosis is now widely believed to be in fact due to too loose a crimp which allows the prosthesis to continually rub and gradually erode the long process. Although erosion is commonly seen with crimped wire, it can also be seen with a variety of other prostheses [17, 21]. The author has noted several cases where loose prostheses have caused notching of the lateral surface of the incus and the recurrence of a fluctuating conductive hearing loss. Early intervention with tightening and use of bone cement is recommended before the incus completely necroses. The author has also had several cases where the oval window fenestrations were too small, causing rocking of the prosthesis and upward pressure also leading to erosion of the incus, lateral displacement, or even extrusion of the prosthesis through the tympanic membrane. Char or burn of the incus adjacent to a crimped wire is not uncommonly encountered and poses a theoretical risk that thermal injury due to laser may lead to incus necrosis.

**Fig. 1:**
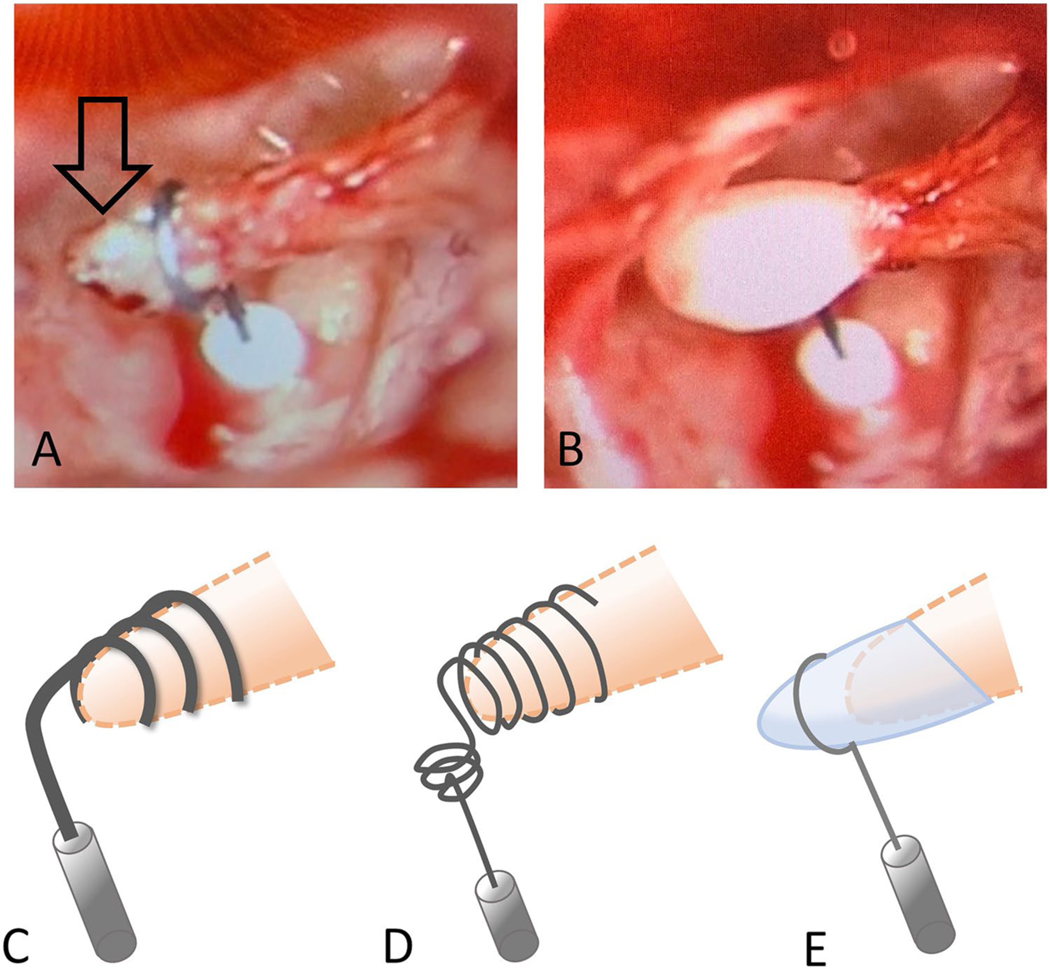
Solutions for incus erosion. (**A**) Arrow points to the incus erosion, and the prosthesis is placed higher up the incus long process. (**B**) Bone cement is then used to stabilize the prosthesis. Alternatively, a Megerian Nitinol Stapes Replacement prosthesis (**C**) or Kraus K-Helix prosthesis (**D**) can be utilized. (**E**) Hydroxyapetite bone cement can also be used to rebuild the shortened incus long process onto which the prosthesis can be secured

For repair, in the case of incus erosion, replacement with an appropriately sized prosthesis higher up the incus handle may be possible. This can pose several problems including re-erosion, improper angle of the prosthesis in order to reach the oval window, or the prosthesis may have a tendency to rest on the facial nerve [14]. In this case, the use of a wire that can be bent away from the facial nerve is an advantage over non-bendable materials. Also, the tapered end of the eroded incus may not allow for prosthesis to attach securely. The use of hydroxyapetite bone cement to rebuild the shortened incus or to stabilize a prosthesis can be implemented (Fig. 1; case 1, case 2), and the outcomes are very good (72–78% within 10 dB) with either method [21, 25, 26]. In the case of a short incus, a Megerian Nitinol Stapes Replacement prosthesis (Fig. 1C) or a Kraus K-Helix prosthesis (Fig. 1D) or may be suitable. Alternatively, a malleus to oval window prosthesis may be utilized.

### Adhesions

Adhesions and fibrous bands are common. The incus or prosthesis can be fixed by dense fibrous tissue [11]. The rate of adhesions is higher with stapedectomy versus stapedotomy (7.9% vs. 20.6%) and is attributed to a less traumatic approach with stapedotomy [11]. Atraumatic lysis may be more easily achieved with a laser, particularly around the oval window. One study reported improved revision outcomes from 70 to 91% (ABG within 10 dB) with the implementation of the argon laser during revision surgery [13]. Although a more recent study, comparing the energy level of the laser and use of the laser versus not did not find an effect on outcome of hearing improvement after revision stapedectomy [27]. The author routinely utilizes the argon laser to lyse adhesions and strongly feels that, at primary surgery, care should be taken not to disrupt or traumatize the mucosa surrounding the oval window or promontory that can lead to fibrosis of the prosthesis or incus binding it to the promontory or tympanic membrane.

### Oval Window Problems

A fairly common occurrence and need for revision are osseous regrowth at the stapedectomy or oval window fenestra 30.2% [11]. Rarely, oval window membrane lateralization or bony regrowth can displace the prosthesis out of the vestibule [11, 17]. A fenestra that is too small can bind the prosthesis and may require drilling or use of a laser to widen the fenestration. Obliterative otosclerosis that greatly thickens the footplate is rare and can be a daunting experience when deep drilling cannot identify a vestibule. Inexperienced surgeons should probably abandon these cases and recommend amplification.

### Ankylosis of the Malleus or Incus

It is important to palpate the entire ossicular chain as one cause for persistent conductive hearing loss may be from a fixed malleus and/or incus, either congenitally or from bone dust or fragments at the primary surgery. Although this occurs rarely (less than 4%) [11, 14], it portends a poorer hearing outcome with a high failure rate of 37.5% [11]. In the case of malleus head fixation, if a bony bridge can be reached, it can be freed with the use of a laser or a drill, but often, this is not possible as the bridge of bone is anterior and superior to the head of the malleus. In such a case, the head can be removed with a malleus nipper, and a malleus attachment prosthesis employed after the incus is removed.

### Perilymphatic Fistula

Perilymphatic fistula (PLF) can be encountered incidentally during revision surgery (case 3) or during revision surgery being performed because of new onset of vertigo or persistent vertigo after a primary surgery that was otherwise uneventful. Typically, one would not operate on sensorineural hearing loss after stapedectomy, unless there was symptomatic vertigo concerning for PLF. In one study, the incidence of delayed vertigo after stapedectomy was 0.5% [28]. Among nine patients taken for exploration, the presence of PLF was only identified in three, although all underwent fibrin glue repair with resolution of vertigo. This and other studies suggest that the presence of PLF is likely underestimated [13]. Another study reported that PLF accounted for 9% of revision stapes, and all of them were associated with vertigo [15]. These data suggest that, if a patient experiences vertigo (with or without sensorineural hearing loss), suspicion for PLF should be raised and exploration be performed. If a tissue seal was not performed at the primary surgery, then suspicion for PLF should be heightened in a patient with vertigo. Although early postoperative hearing may not be significantly changed, there may be delayed sensorineural hearing loss and decreased discrimination [17].

### Reparative Granuloma

**Fig. 2:**
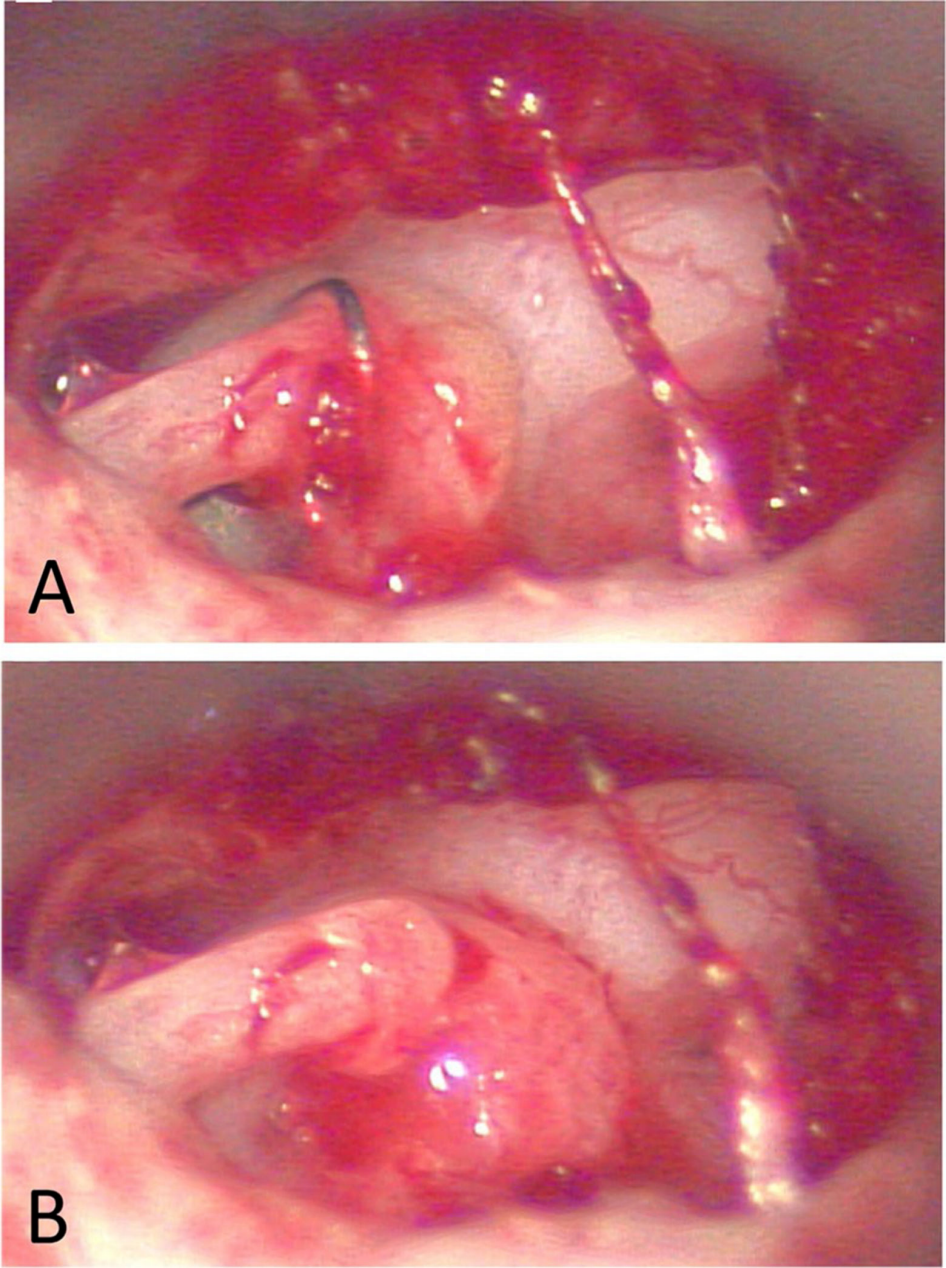
Post-stapedotomy reparative granuloma following use of acellular porcine small intestinal submucosa. **A** Intraoperative view of reparative granuloma surrounding Biodesign (porcine graft) and bucket-handle prosthesis. **B** Removal of bucket-handle prosthesis from incus and off footplate reveals granuloma centered around Biodesign and oval window. (Reprinted from Ghazi et al. Am J Otolaryngol. 2021;42(3):102,933, with permission from Elsevier) [30•]

This is a rare condition that occurs usually 7–15th-day post-operatively after a stapedectomy [29]. It was highly associated in the past with manufactured gelfoam-wire prostheses, but since they were taken off the market, this is an even rarer occurrence. Some are thought to be due to powder on gloves, but this has not been proven [29]. A recent report involved a granuloma after acellular porcine small intestinal submucosa was used to seal the piston at the oval window (Fig. 2) [30•]. Patients present with worsening sensorineural hearing loss after initial improvement and may be accompanied by worsening imbalance. On examination of the ear, there will be fullness and a darkened area in the posterior mesotympanum where the granuloma typically covers the incus and surrounds the prosthesis down to the oval window. In a survey done of stapes surgeon, it was determined that the incidence in stapedectomy cases was 0.1%, and in stapedotomy, the incidence declined to 0.07% [31]. Management of these cases is controversial. One school of thought is to emergently return the patient to the operating room and to remove the granuloma and replace the prosthesis with a new one, combined with concurrent antibiotics and high dose steroids [29, 32]. The other consideration is to simply treat the patient with corticosteroids based on experience that the outcomes will be the same [33]. Since this is a rare condition in the modern era of stapes surgery, it is still unknown which method of management is preferable.

### Case Examples

Here, we provide a few examples of commonly described problems along with their solutions.

### Case 1 (Fig. 3), Dislodged Prosthesis and Shortened Incus

The twenty-three-year-old male had undergone two prior stapedotomies and had a recurrent maximum conductive hearing loss in the right ear with the wire protruding through the tympanic membrane. He underwent a revision at which time the dislodged prosthesis was removed, and the lenticular process of the incus was found to be necrosed and too short for a traditional piston. The fenestration of the footplate was seen to be too small, so it was enlarged with an argon laser. A Kraus K-Helix prosthesis (Fig. 1D) was then used, and its attachment was coated with bone cement. No post-operative complications occurred and he had excellent post-op hearing improvement.

### Case 2 (Fig. 4), Incus Erosion, Rebuilding of Incus with Bone Cement

Sixty-six-year-old male underwent prior bilateral stapedotomies and a revision on the left side. He recently developed a further drop in left hearing with vertigo, imbalanced, and a profound mixed loss with 60% speech discrimination. He experienced vertigo undergoing a tympanometry test. He underwent left middle ear exploration with monitored anesthesia and was found to have incus necrosis with the piston medially displaced into the vestibule. A laser was used to lyse adhesion, and the piston was slowly lifted out of the vestibule. There was a medially displaced portion of the footplate that was carefully removed with a small hook. The incus was short, so hydroxyapetite bone cement was used to extend the incus, and then, a new nitinol prosthesis was placed, and additional bone cement was applied to further secure it (Fig. 1E). The patient had total resolution of his dizziness, and hearing was significantly improved with return of his speech discrimination to 96%.

### Case 3 (Fig. 5), Lenticular Process Fracture, Medial Displacement of Prosthesis, and Perilymph Leak

A forty-two-year-old female had undergone bilateral stapedotomies many years before. She had a sudden drop in hearing in the right ear that showed a severe mixed loss with good speech discrimination. Upon exploration under monitored anesthesia, the lenticular process was found to be fractured, and the entire piston of the prosthesis had been displaced medially into the vestibule with an obvious perilymph leak. An argon laser was used to lyse all adhesions, so that the wire and footplate could be seen clearly. The Shepherd’s crook was used to lift the prosthesis out of the vestibule, and the patient monitored for dizziness which did not occur. A new nitinol prosthesis was hooked onto the remaining incus and crimped with a laser. Morselized ear lobe fat was placed around the prosthesis, and bone cement was then applied to stabilize the prosthesis on the incus. The patient had an uneventful recovery with good closure of the air–bone gap.

### Case 4 (Fig. 6), Tympanic Membrane Perforation, Displaced Prosthesis, and Obliterative Otosclerosis

A fifty-one-year-old female with prior stapedotomies in both ears had excellent results until 14 years later when, after aerotitis (pain and pressure on decent), her hearing declined. A few months later, she was noted to have a perforated right tympanic membrane with exposed prosthesis. Her right Rinne was negative (bone greater than air conduction). Upon exploration, the prosthesis was found displaced off of the footplate, and the incus had a notch where the loose Shepherd’s crook was still attached. The piston was not in the vestibule and was removed. The footplate was obliterated with new overgrowth of otosclerosis. A low rpm drill was used to fenestrate the footplate, and a new nitinol prosthesis was secured with a laser. Bone cement was applied over the crimp after small pieces of ear lobe fat were placed around the piston. Excellent post-op hearing was achieved.

## Conclusions

With improvements to stapedectomy, the rate of success of primary surgery is excellent. The closure of the ABG may not remain so over time, however. The mean time between primary and revision operations is typically 7–11 years [11, 14, 18]. Typical time to the next re-revision is shorter, 2–4 years [11, 14]. The most common indication for revision surgery is persistent or acquired conductive hearing loss, although sometimes, it is for vertigo.

Revision surgery, however, continues to prove challenging with the problems that are encountered and with the overall success rate which is much less than that of primary surgery, even in the most experienced hands. The overall closure of the ABG is 45–71% to within 10 dB and 75.7–86.3% to within 20 dB [11, 13, 17, 34, 35]. Unsurprisingly, the outcome is worse by 10–20 dB with each successive revision [13, 17]. Furthermore, risk of developing a dead ear is slightly higher at ∼2% [14].

The vast majority of cases have a prosthesis displacement with or without incus erosion. These cases can be repaired by various ways as aforementioned. The good news is that the repair of these problems fair well. The same cannot be said for malleus/incus ankyloses and obliterative otosclerosis which routinely have poor outcome but are encountered less often.

How do we maximize the success of revision surgery? First, it is important to carefully evaluate the reasons for re-operating. Reasons include a persistent or acquired conductive hearing loss of > 20 dB, a clear prosthesis problem seen on exam or CT, and vertigo due to suspected perilymphatic fistula or too long a prosthesis. Revision stapedectomy should not be performed within 6 weeks of primary surgery, for sensorineural hearing loss in the absence of vertigo, obliterative otosclerosis seen on CT not amenable to drill out, round window obliteration, and the presence of the third window such as a semicircular canal dehiscence. The surgeon must also understand his/ her own ability to anticipate and manage the intraoperative findings as described earlier. Lastly, it is important that the patient is educated on realistic expectations with revision outcomes.

## Figures and Tables

**Fig. 3:**
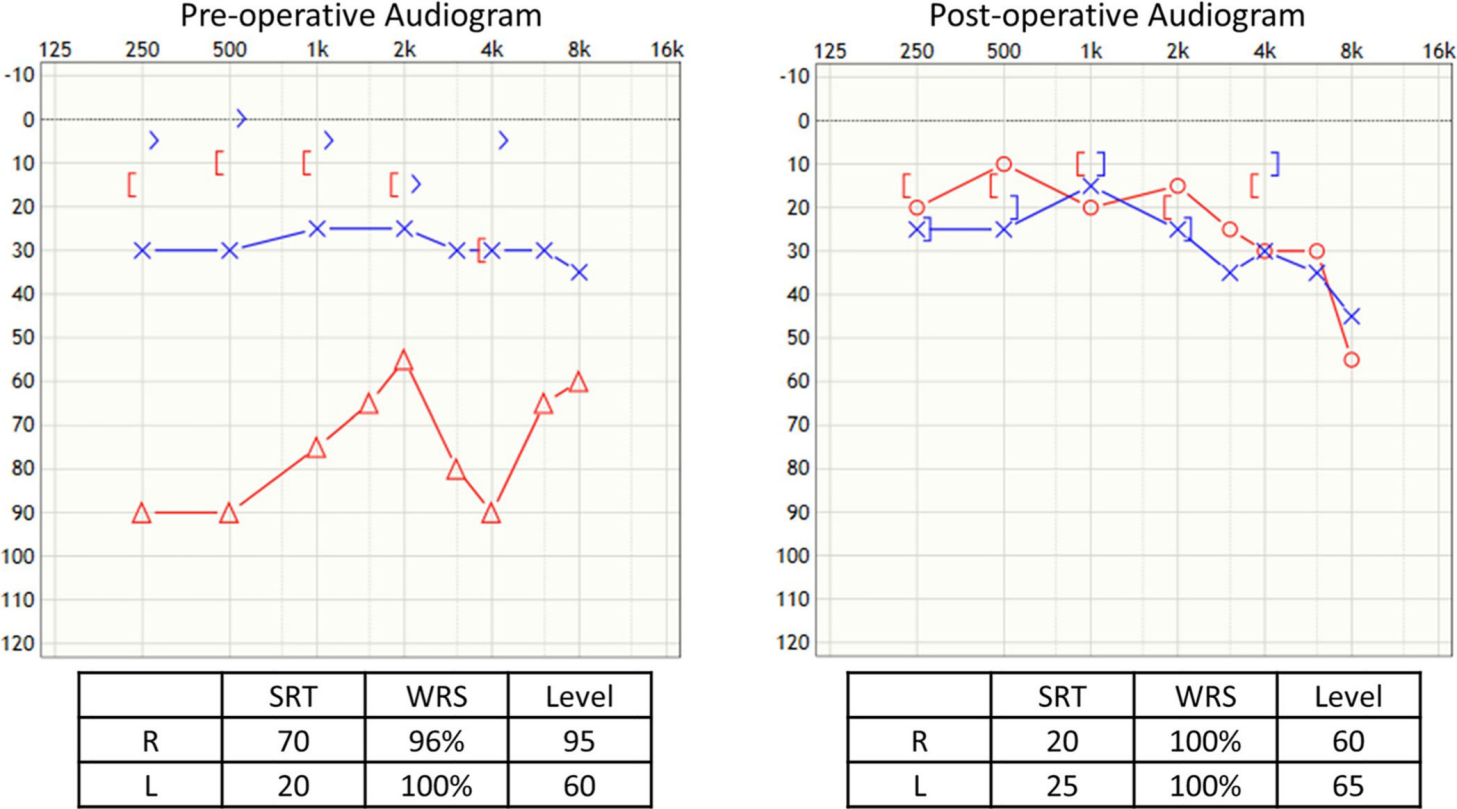
Case 1, dislodged prosthesis and shortened incus

**Fig. 4:**
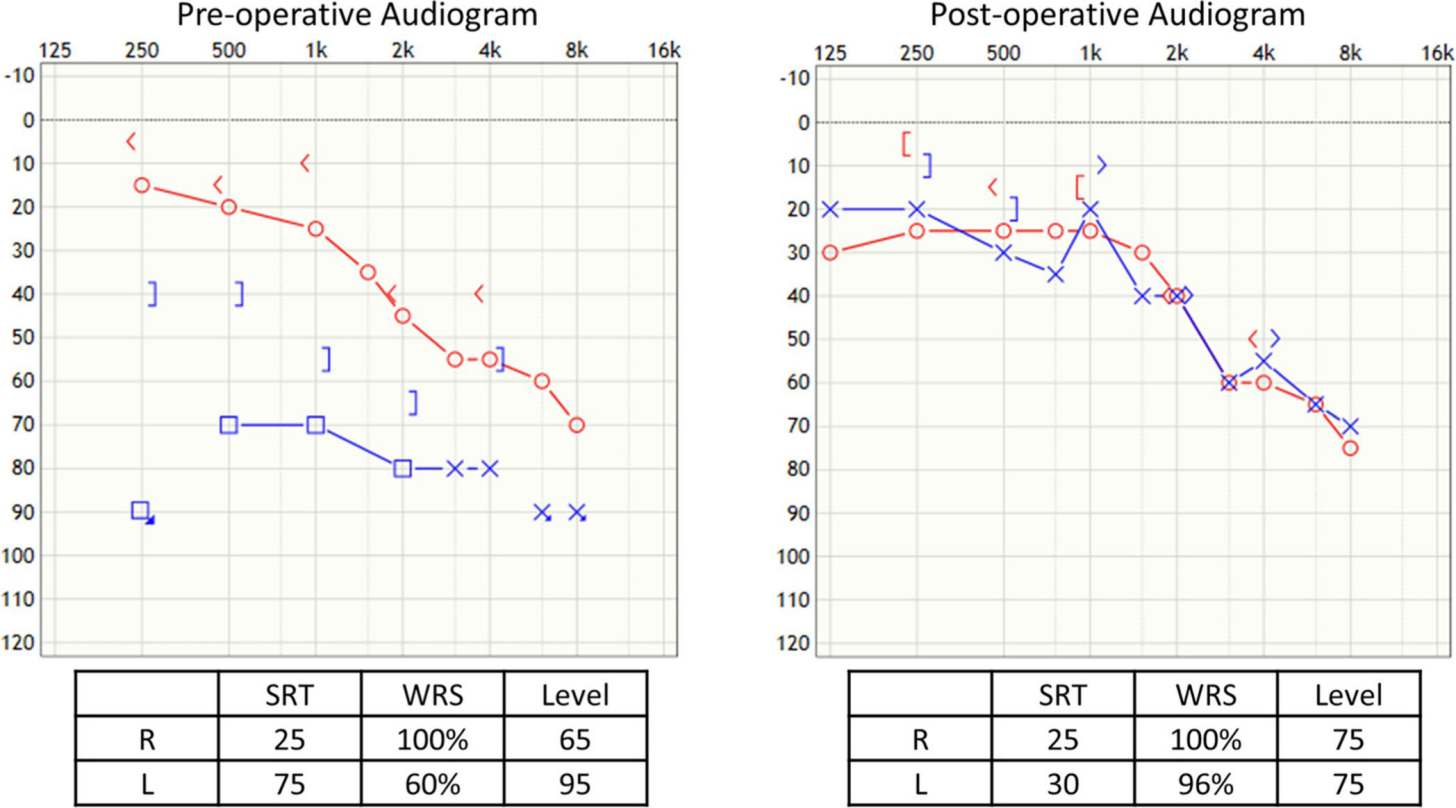
Case 2, incus erosion, rebuilding of incus with bone cement

**Fig. 5:**
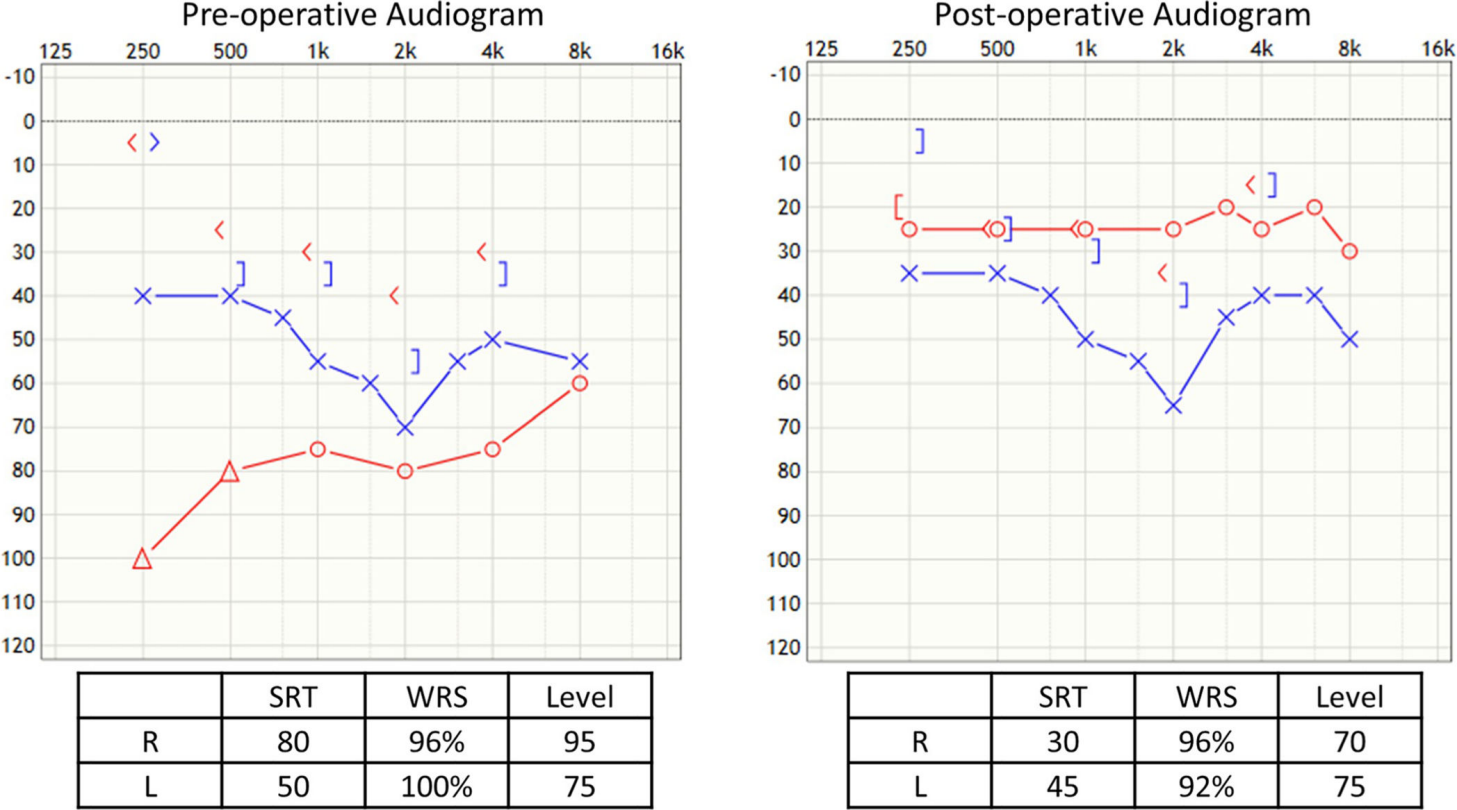
Case 3, lenticular process fracture, medial displacement of prosthesis, and perilymph leak

**Fig. 6:**
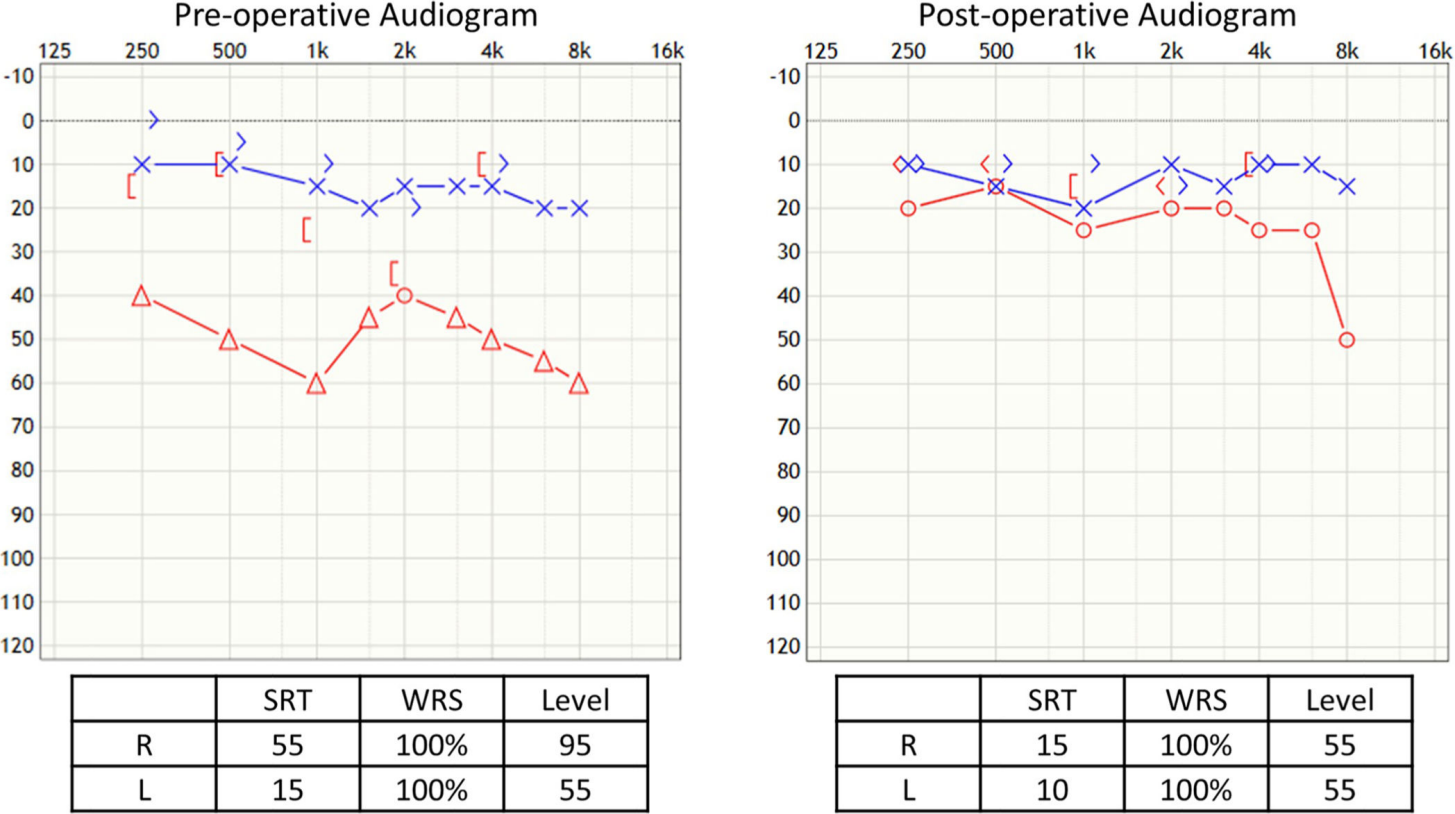
Case 4, tympanic membrane perforation, displaced prosthesis, and obliterative otosclerosis
